# 470. Clinical Effectiveness of The Monoclonal Antibody Combination Tixagevimab-Cilgavimab (T/C) as Pre-Exposure Prophylaxis (PReP) of COVID-19 among Patients (pts) with Moderate to Severe Immunocompromised (IC) Conditions across a Large U.S. Healthcare System: A Propensity Score-Matched Retrospective Cohort Study

**DOI:** 10.1093/ofid/ofad500.540

**Published:** 2023-11-27

**Authors:** Ghady Haidar, Justin Ludwig, Donald M Yealy, Haley Camacho, Tina Chinakarn, Kevin Kip, Stephen Koscumb, Oscar Marroquin, Tami Minnier, Graham M Snyder, Chelsea Woodworth, Cátia Ferreira, Lisa Glasser, Kathleen Heil, Andrew Lee, Carla Talarico, Sudhir Venkatesan, Sylvia Taylor, Erin K McCreary, John W Mellors

**Affiliations:** University of Pittsburgh School of Medicine, Pittsburg, PA; Office of Quality and Clinical Research Innovation, University of Pittsburgh Medical Center, Pittsburgh, PA, USA, Pittsburgh, Pennsylvania; University of Pittsburgh Medical Center, Pittsburgh, PA, USA, Pittsburgh, Pennsylvania; Office of Quality and Clinical Research Innovation, University of Pittsburgh Medical Center, Pittsburgh, PA, USA, Pittsburgh, Pennsylvania; Office of Quality and Clinical Research Innovation, University of Pittsburgh Medical Center, Pittsburgh, PA, USA, Pittsburgh, Pennsylvania; UPMC Health Services Division, University of Pittsburgh Medical Center, Pittsburgh, PA, USA, Pittsburgh, Pennsylvania; University of Pittsburgh Medical Center, Pittsburgh, PA, USA, Pittsburgh, Pennsylvania; University of Pittsburgh Medical Center, Pittsburgh, PA, USA, Pittsburgh, Pennsylvania; University of Pittsburgh Medical Center, Pittsburgh, PA, USA, Pittsburgh, Pennsylvania; University of Pittsburgh, Pittsburgh, PA; University of Pittsburgh Medical Center, Pittsburgh, PA, USA, Pittsburgh, Pennsylvania; Vaccines and Immune Therapies, BioPharmaceuticals Medical, AstraZeneca, Wilmington, DE, USA, Wilmington, Delaware; Vaccines and Immune Therapies, BioPharmaceuticals Medical, AstraZeneca, Wilmington, DE, USA, Wilmington, Delaware; Vaccines and Immune Therapies, BioPharmaceuticals Medical, AstraZeneca, Wilmington, DE, USA, Wilmington, Delaware; Medical and Payor Statistics, BioPharmaceutical Business Unit, AstraZeneca, Cambridge, UK, Cambridge, England, United Kingdom; Vaccines and Immune Therapies, BioPharmaceuticals Medical, AstraZeneca, Gaithersburg, MD, USA, Gaithersburg, Maryland; Medical and Payer Evidence Statistics, BioPharmaceutical Medical, AstraZeneca, Cambridge, UK, Cambridge, England, United Kingdom; Medical Evidence, Vaccines and Immune Therapies Unit, AstraZeneca, Cambridge, UK, Cambridge, England, United Kingdom; UPMC, Pittsburgh, PA; University of Pittsburgh School of Medicine, Pittsburg, PA

## Abstract

**Background:**

T/C authorization in the US for PrEP of COVID-19 in IC individuals was initially based on a randomized trial (PROVENT). However, < 5% of enrollees in PROVENT were IC. We sought to assess real-world effectiveness of T/C PrEP among IC pts.

**Methods:**

We conducted a retrospective, observational, propensity-score matched cohort study at UPMC from Jan 1, 2022, to Mar 31, 2023. We assessed effectiveness at ≤ 6 months of T/C 600 mg (as initial dose, or as 300 mg then 300 mg ≤ 3 months later) against chart confirmed COVID-19 hospitalizations and COVID-19–related deaths among IC pts.

**Results:**

Before matching, there were 2931 T/C vs 157,225 non-T/C pts. After matching, 2301 matched pairs remained (78.5% vs 1.8% of all eligible T/C vs non-T/C pts). Most pts had moderate/severe IC conditions (**Table 1**). During the study period encompassing circulating variants both susceptible and resistant to T/C, there were 15 vs 18 COVID-19 hospitalizations across 377,832 person-days in T/C vs non-T/C pts (**Table 2**). Thus, 0.72% vs 0.87% of T/C exposed vs unexposed pts were hospitalized for COVID-19 (HR 0.833, 95% CI 0.39 – 1.75, p = 0.73) (**Table 2; Figure 1**). There was no difference in effectiveness by T/C dosing pattern (**Table 2**). Effectiveness was numerically higher in the BA.5 period, although there were few events in other variant eras (**Table 2**). There was a non-significant 50% lower risk of hospitalization in T/C patients with solid tumors or autoimmune conditions, and no significant difference by exposure status in patients with organ transplant or hematologic cancer (**Table 2**). There were 5 COVID-19 ICU admissions per exposure group, and 2 inpatient COVID-19–related deaths (1 per group) (**Table 3**). Limitations included overall low event rate limiting power and an open healthcare system leading to possibility of missing hospitalizations due to pts seeking care elsewhere.

**Figure:**
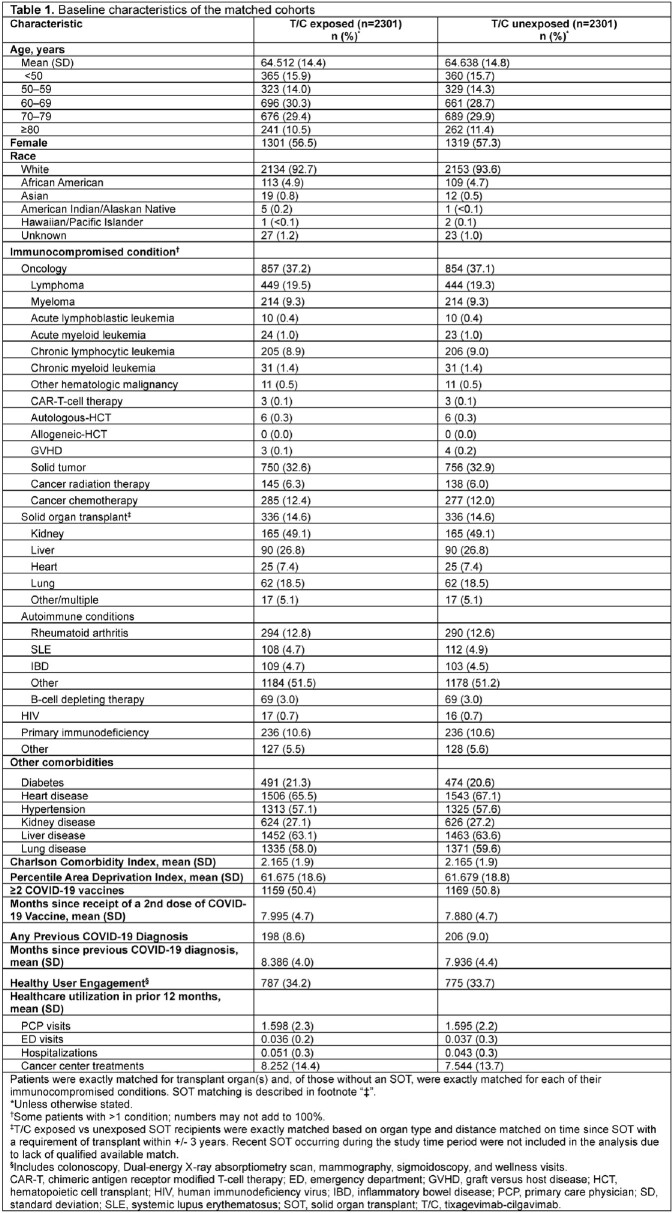

**Figure:**
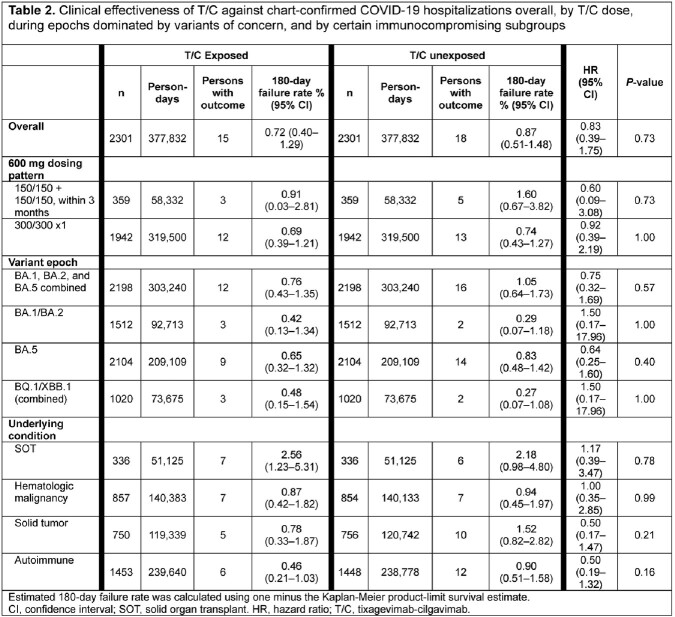

**Figure:**
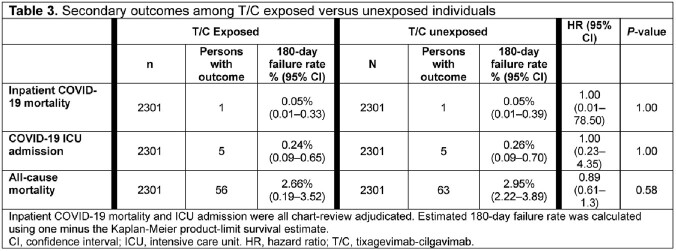

**Conclusion:**

The overall low number of chart-confirmed COVID-19 hospitalizations for the entire cohort limited our ability to detect a statistical difference between T/C exposed vs. unexposed patients. Possible clinical benefit of T/C is suggested for solid tumor and autoimmune patients, although larger studies will need to confirm this observation.

**Figure 1.**
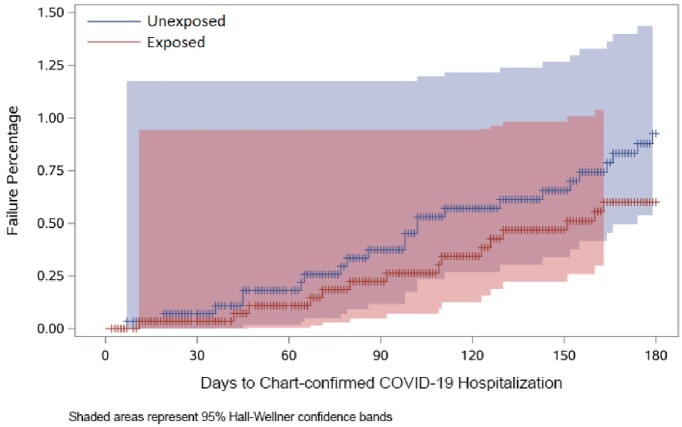
Time-to-COVID-19 hospitalization (chart-review confirmed) for entire period among T/C exposed (red) vs unexposed (blue) patients.

**Disclosures:**

**Ghady Haidar, MD**, Allovir: Grant/Research Support|AstraZeneca: Advisor/Consultant|AstraZeneca: Grant/Research Support|Karius: Advisor/Consultant|Karius: Grant/Research Support|NIH: Grant/Research Support **Graham M. Snyder, MD, SM**, Infectious Diseases Connect: Advisor/Consultant **Cátia Ferreira, PhD**, AstraZeneca: Employee **Lisa Glasser, MD**, AstraZeneca: Stocks/Bonds **Kathleen Heil, RN, BSN**, AstraZeneca: Employee **Andrew Lee, MSc**, AstraZeneca: Employee **Carla Talarico, PhD, MPH**, AstraZeneca: Stocks/Bonds **Sudhir Venkatesan, MPH, PhD**, AstraZeneca: Employee **Sylvia Taylor, PhD, MPH, MBA**, AstraZeneca: Stocks/Bonds **Erin K. McCreary, PharmD**, Abbvie: Advisor/Consultant|Ferring: Advisor/Consultant|GSK: Honoraria|La Jolla (Entasis): Advisor/Consultant|LabSimply: Advisor/Consultant|Merck: Advisor/Consultant|Shionogi: Advisor/Consultant|Shionogi: Honoraria **John W. Mellors, MD**, AstraZeneca: Grant/Research Support|Gilead Sciences: Grant/Research Support

